# A Growing Two-Decade-Old True Left Ventricular Aneurysm: A Case Report

**DOI:** 10.7759/cureus.18792

**Published:** 2021-10-14

**Authors:** Alexander Kong, Ramses Ramirez Damera, Alberto Perez Buitrago, Hiep C Nguyen, Sayed T Hussain

**Affiliations:** 1 Internal Medicine, University of Central Florida-HCA Healthcare Graduate Medical Education (GME), Orlando, USA; 2 Internal Medicine, University of Medicine and Health Sciences, Basseterre, KNA; 3 Cardiothoracic Surgery, University of Central Florida College of Medicine, Orlando, USA; 4 Cardiology, University of Central Florida College of Medicine, Orlando, USA

**Keywords:** left ventricular aneurysm, true aneurysm, myocardial infarction, mechanical complication, late complication, cardiac surgery, aneurysmectomy

## Abstract

Left ventricular aneurysms (LVA) occur after an infarcted area of the myocardium necrotizes, fibroses, and expands, forming a dyskinetic cavity. Most ventricular aneurysms are asymptomatic and go unrecognized unless found incidentally. Symptoms commonly reported include angina, heart failure, syncope, and even sudden cardiac death. Late complications from left ventricular aneurysms are infrequently reported. This case reports an elderly woman who presented with new-onset angina from an expanding 18-year-old true left ventricular aneurysm that was successfully treated with surgical repair.

## Introduction

True left ventricular aneurysms (LVA) most commonly occur after an infarcted area of the myocardium necrotizes, fibroses, and expands, forming a dyskinetic cavity. True left ventricular aneurysms are surrounded by necrotic tissue, muscle, and pericardium, whereas false ventricular aneurysms (pseudoaneurysms) contain pericardial tissue only and are thereby at increased risk of rupture [1]. The incidence of LVA has been decreasing steadily since major advancements in coronary revascularization after acute myocardial infarction, particularly in the last decades, from a historic 30%-35% to about 8%-15% in the 1990s, to a current incidence of less than 5% [2,3]. Most true LVA, especially small ones, are asymptomatic and go unrecognized unless found incidentally during routine cardiovascular imaging [1]. Symptoms commonly reported include angina, heart failure, syncope, and even sudden cardiac death [2]. A high level of suspicion is imperative since life-threatening complications may occur unpredictably at any given time. These include heart failure and angina, due to decreased effective cardiac output and myocardial ischemia from increased oxygen demand; ventricular arrhythmias secondary to increased myocardial wall stretch and ischemia; and thromboembolism from mural thrombus formation [2]. Ventricular free wall rupture in true LVA rarely happens due to the dense fibrosis of the aneurysmal wall [4]. This case reports an elderly woman who presented with acute complications from an 18-year-old large true LVA.

## Case presentation

A 73-year-old woman presented with three days of progressively worsening sharp non-radiating reproducible left-sided thoracic pain exacerbated by movement of the ipsilateral upper extremity. Her past medical history was significant for a myocardial infarction two decades prior with resultant ischemic cardiomyopathy and congestive heart failure with moderately reduced left ventricular ejection fraction and a biventricular pacemaker and defibrillator in place. Initial examination revealed normal vital signs, non-distended neck veins, and left-sided chest wall tenderness to palpation. Electrocardiography displayed a ventricular-paced rhythm and left bundle branch block not meeting the Sgarbossa criteria for ischemia. Three serial troponin I assays were negative. Chest radiograph showed dual chamber pacer wires in place without cardiomegaly, pulmonary edema, or pleural effusions. Biventricular pacemaker interrogation revealed no arrhythmic events but indicated the need for battery replacement. Her symptoms were attributed to noncardiac musculoskeletal chest pain, and she was discharged home with plans for elective pacemaker battery replacement.

**Figure 1:**
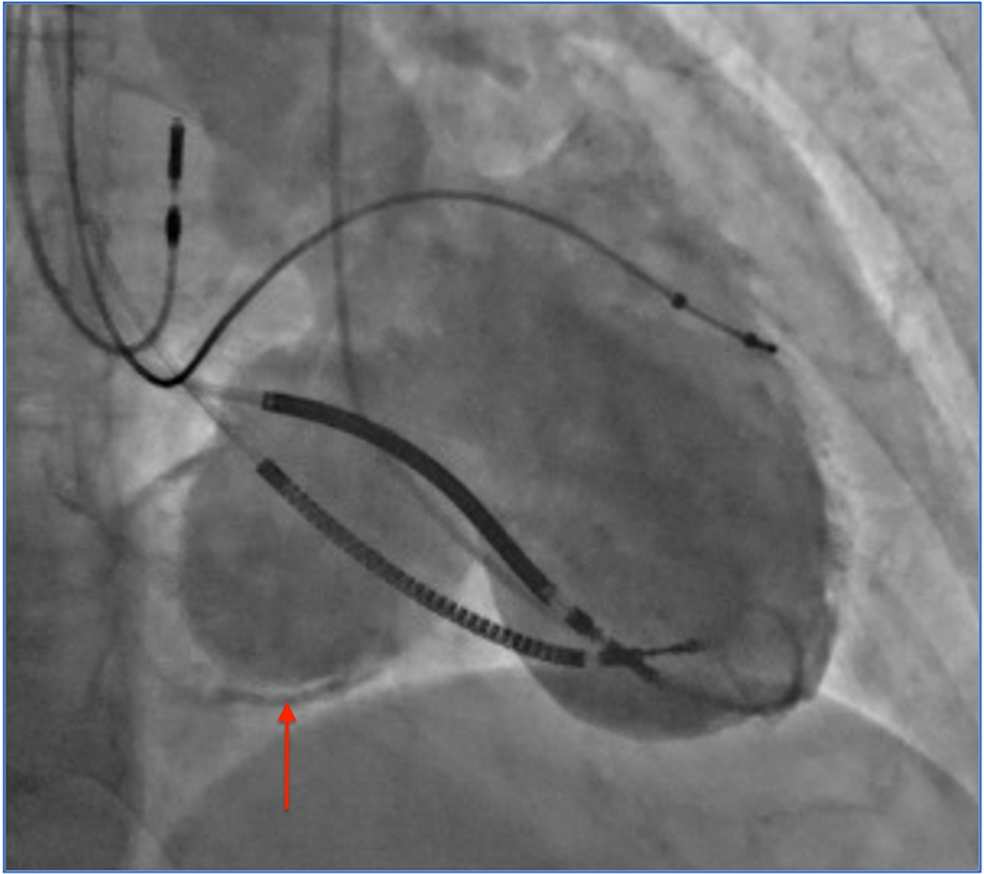
Left ventriculography showing a large left ventricular infero-basal aneurysm with a 2.8 cm neck.

**Figure 2:**
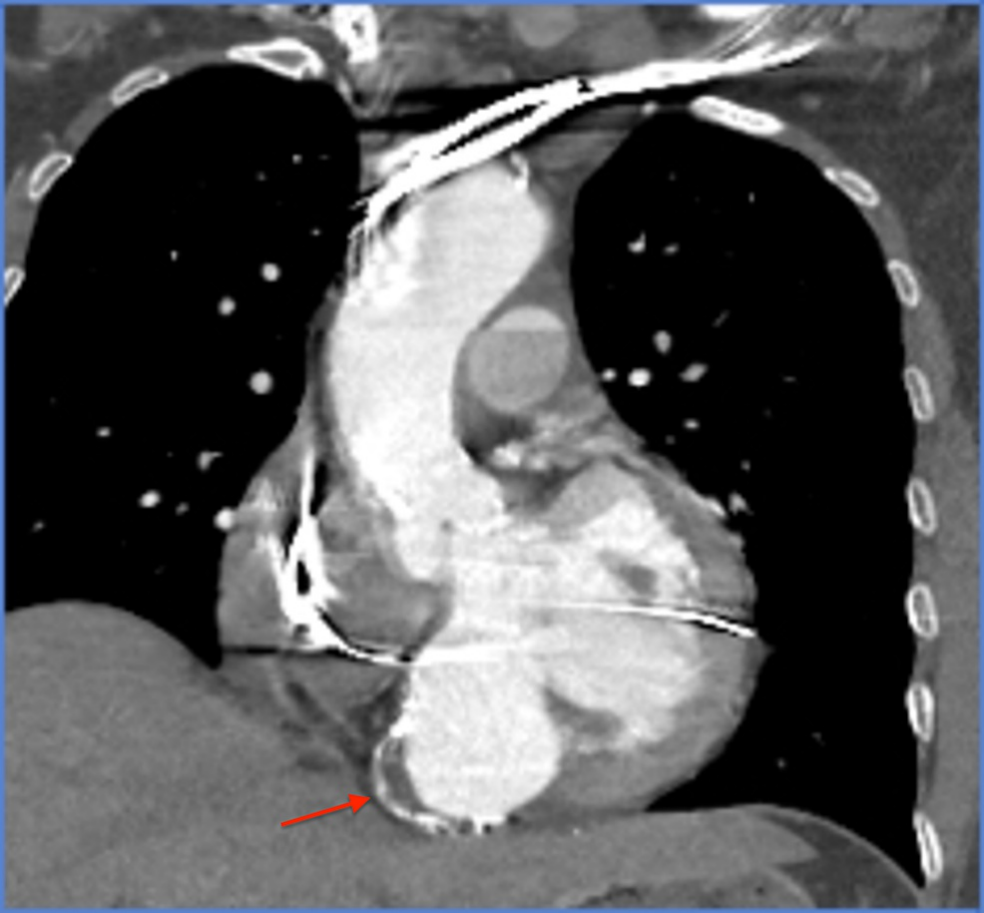
Cardiac computed tomography with angiography of the chest showing a large left true ventricular aneurysm.

**Figure 3:**
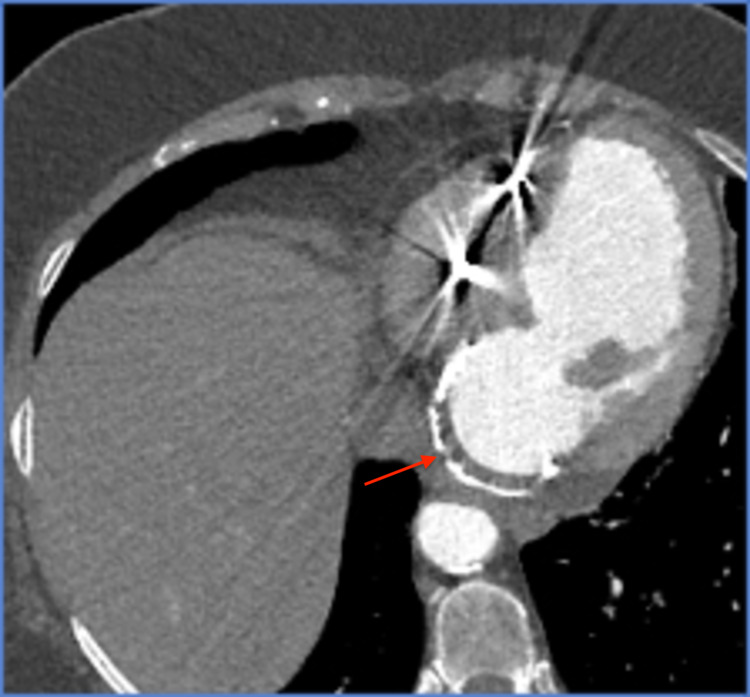
Cardiac computed tomography with angiography showing a large 46 x 45 x 47 mm infero-basal aneurysm with a chronic thrombus and calcified wall.

**Figure 4:**
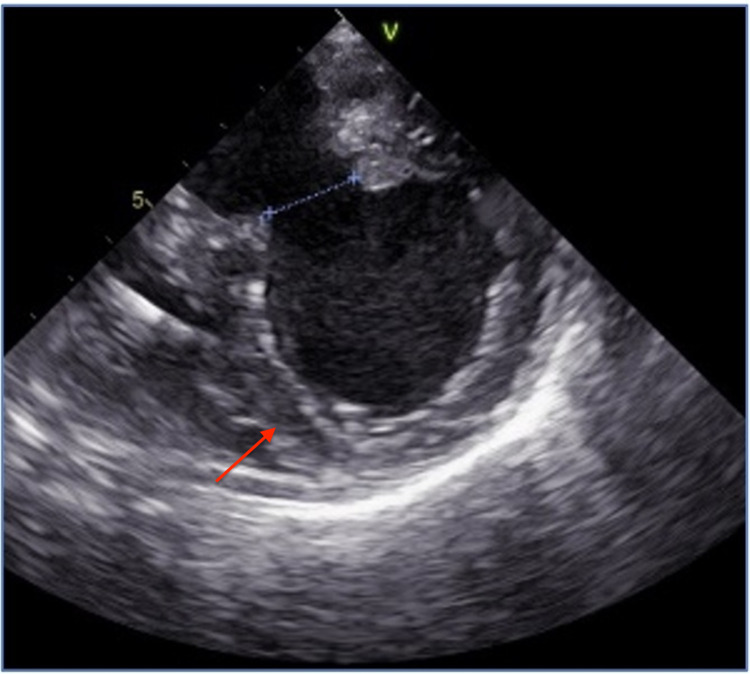
Transesophageal echocardiogram showing a large left ventricular aneurysm.

Two weeks later, the patient presented with dyspnea, orthopnea, and retrosternal chest pain. On physical examination, the patient was afebrile, and she had a blood pressure of 172/64 mmHg, pulse rate of 67 beats/minute, and respiration rate of 18 breaths/minute. Her oxygen saturation was 99% on 3 L of oxygen per minute. Cardiac examination revealed non-distended neck veins, normal heart sounds, diminished bilateral lung sounds, and mild bilateral pedal edema. Electrocardiogram was similar to the prior, and serial cardiac enzymes were normal. The brain natriuretic peptide level was elevated at 2,042 pg/mL. Chest radiograph showed increased vascular markings and cardiomegaly. Immediate intravenous loop diuretics and noninvasive positive pressure ventilation were initiated with symptomatic improvement. In the setting of unstable angina with decompensated heart failure, urgent cardiac catheterization was performed, revealing a totally occluded right coronary artery, normal left coronaries, and a calcified, well-demarcated, large left ventricular infero-basal aneurysm with a 28 mm neck opening (Figure 1). Cardiac computed tomography with angiography (Figures 2 and 3) and transesophageal echocardiography (Figure 4) showed a 46 x 45 x 47 mm aneurysm originating just at the takeoff of the posterior mitral valve leaflet (increased in size when compared with prior images) and severe mitral regurgitation. The patient underwent surgical mitral valve repair, aneurysmectomy, and pericardial patching. Left ventricular ejection fraction improved from 30%-35% to 40%-45% postoperatively. Following the initial recovery period, the patient reported better exercise tolerance and improved quality of life, and she did not require further heart failure or angina-related hospitalizations to date.

## Discussion

This patient’s history reflects the development of a large true left ventricular infero-basal aneurysm, likely due to total right coronary artery occlusion as a result of her prior non-revascularized coronary syndrome two decades ago. Inferior-posterior LVA occur in only 5%-10% of cases, while the most common are apical and anteroseptal aneurysms from complete proximal left anterior descending artery occlusion [5]. 

As a long-term complication of her ischemic cardiomyopathy and LVA, the patient had developed symptomatic heart failure, New York Heart Classification stage II, with severely reduced ejection fraction requiring implantable cardiac defibrillator and cardiac resynchronization therapy. Moreover, due to the aneurysm’s close proximity to the mitral valve apparatus, the patient developed severe mitral regurgitation. 

In her initial presentation to the hospital, the patient’s symptoms were consistent with musculoskeletal chest pain. Clinically, the patient did not have evidence of overt heart failure, ongoing myocardial ischemia, syncope, or other immediately life-threatening complications. Hence, she was deemed to be safe for discharge and close outpatient follow-up for elective mitral valve and aneurysm repair. Two weeks later, the patient returned with symptoms highly suggestive of an acute coronary syndrome and was therefore directed for urgent percutaneous coronary angiography, which did not evidence new significant obstructive disease but showed a large true left ventricular aneurysm with a large neck. Although pacemaker dysfunction as a potential cause of heart failure exacerbation could have explained the patient’s symptoms, in hindsight, she clinically improved only after the surgical repair of her LVA and before elective pacemaker battery exchange. LVA expansion is associated with increased myocardial oxygen demand and subsequent ischemia, which could explain the anginal symptoms in this patient. 

Expansion of her inferior-posterior LVA ensued despite the presence of extensive collateral circulation of her totally chronically occluded right coronary artery and strict compliance with guideline-directed medical therapy. The exact timeline of her LVA progression remains unknown due to the absence of imaging studies for comparison in the interim. However, the 18-year stability of her symptoms up to presentation and change in size when compared with prior images suggest that late aneurysmal expansion occurred.

Intuitively, surgery could have been offered to the patient at an earlier time since her LVA was known to be symptomatic for many years. Nonetheless, medical management remains the first-line treatment strategy for LVA-related complications, while surgery is reserved for the treatment of refractory symptoms per the most current guidelines [3,6]. Independent risk factors for increased in-hospital mortality after aneurysm repair include surgery in the preceding decade and heart failure, with better outcomes if the reason for repair is a thromboembolic disease or angina [7]. Since the patient did not suffer from the latter until this presentation, earlier aneurysm surgical repair for heart failure alone may have been counterproductive and resulted in worse outcomes.

Recent studies suggest that an endocardial surgical approach provides the best outcomes in LVA repair [7,8]; however, a ventriculostomy was performed instead given the aneurysm’s close proximity to the mitral valve chordae tendineae. 

This case emphasizes the importance of knowing that LVA may be stable for many years and can acutely worsen, causing life-threatening complications. In this setting, surgical repair can improve ejection fraction, exercise tolerance, and quality of life.

## Conclusions

Late ventricular aneurysm complications may occur, even two decades after myocardial infarction. A high index of suspicion is imperative for early recognition and prompt surgical repair, when indicated, which may be life-changing.
